# Decision-Making Styles Shaping College Students’ Sports and Health Consumption Preferences: Behavioral and Neurological Evidence

**DOI:** 10.3390/bs16071099

**Published:** 2026-07-02

**Authors:** Gang Ma, Shengyue Wang, Jialin Fu, Xilin Liu

**Affiliations:** 1 College of Economics and Management, Zhengzhou University of Light Industry, Zhengzhou 450001, China; 2 School of Computer Science and Technology, Harbin Institute of Technology, Weihai 264209, China

**Keywords:** decision-making style, promotion mode, sport and health consumption, college students, functional near-infrared spectroscopy (fNIRS)

## Abstract

To investigate the influence of decision-making styles on college students’ sports and health consumption preferences and the underlying cognitive neural mechanisms, this study recruited 39 college students as participants, adopted a one-factor within-subjects design, and combined behavioral experiments with functional near-infrared spectroscopy (fNIRS). It examined consumption preferences and brain activation characteristics in maximizers and satisficers under three conditions: no promotion, discount promotion, and public welfare promotion. In behavioral terms, college students demonstrated the highest inclination towards public welfare promotions, with discounts being the second most favored, while the no-promotion condition received the lowest preference. Maximizers preferred discount promotion, while satisficers prioritized public welfare promotion. In neural terms, public welfare promotion widely activated the left dorsolateral prefrontal cortex, whereas discount promotion only activated a local region of this cortex. Maximizers showed the strongest activation in the corresponding region under discount promotion, and satisficers exhibited more significant activation in the corresponding region under public welfare promotion. Decision-making styles shaped consumption preferences through depth of information processing and brain activation patterns: maximizers focused on rational calculation and benefit maximization, while satisficers relied on intuitive experience and value perception. These findings provide behavioral and neuroscientific evidence for precision marketing in the sport and health consumption market and the implementation of the national fitness program.

## 1. Introduction

Health represents a fundamental symbol of national prosperity and social progress, and the integrated development of sports and health has become a core pillar for high-quality social undertakings in China (H. Sun et al., 2025; M. Liu, 2025). Driven by the Healthy China Program and the national policy of expanding domestic demand, sports and health consumption has evolved from a supplementary lifestyle to a key engine of consumption upgrading and economic growth (Ma et al., 2025). A series of national initiatives, including the 2025 “Year of Weight Management” campaign (Xu & Ning, 2026) and the Special Action Plan for Promoting Health Consumption, have further guided the market to enrich fitness scenarios and embed health concepts into daily life. To respond to the national call, commercial sports venues have begun to explore public welfare-oriented opening models, such as the more than ten social sports venues in Chongming, Shanghai, that obtained public welfare opening certification in 2024, and the 12 public sports venues in Dalian that implemented the requirement of no less than 35 h of weekly free or low-charge opening in early 2025, which have effectively connected policy orientation with market practice. This mode can be named public welfare promotion in this study, and common modes include discount promotion and no promotion. Under this background, sports and health consumption has become an important carrier for meeting public well-being needs and improving national health literacy, making the market development particularly strategically significant (C. Zhou et al., 2025).

Official national fitness statistics released in 2025 indicated that 38.52% of Chinese residents aged 7 and above regularly participate in physical exercise, and the per capita sports venue area reached 3.11 square meters with the full rollout of the 15 min fitness circle under the Healthy China Initiative (General Administration of Sport of China, 2025). The “Healthy China 2030” strategy receives governmental support through media campaigns that target young adult exercise behaviors (F. Li, 2025). As young consumers equipped with robust health consciousness and proactive spending propensity, college students form a pivotal cohort within sports and wellness consumption. Unlike the general public, college students are more willing to spend their disposable income on sports consumption and are not inclined to cut related budgets, rather than having only a small group of high spenders. Relevant data from the 2025 Insight into College Students’ Sports and Outdoor Consumption shows that the scale of college sports and outdoor consumption is expected to reach 1.3 trillion yuan in 2025, and nearly 77% of college students state that they will not reduce spending on sports-related products and services. Such outstanding group-level consumption readiness and sizable market potential underscore the necessity of exploring college students’ consumption preferences and decision-making logic to facilitate the sound development of the sports and health market.

Sports and health consumption is a complex decision-making process affected by individual cognition, external incentives, and environmental factors (Gould, 1988). Among these influencing factors, internal cognitive traits play a more fundamental and decisive role in shaping personal choices, making it critical to explore how different decision-making styles drive consumption preferences. From a health economics perspective, sports and health consumption is a forward-looking health investment on products and services related to sports (Zhang et al., 2026). Existing studies have extensively examined macro policy, income level (Muurinen, 1982), family structure (H. Li et al., 2024), digital technology (Olga, 2024), and regional differences (Peng et al., 2025) as influencing factors. Some research also confirms the positive effects of physical health (H. Wang et al., 2024), health information-seeking behavior (Kim, 2024), and digital financial literacy (H. Zhou & Xiong, 2025) on sports consumption. However, most studies focus on external and demographic variables, while micro-level research on internal cognitive heterogeneity among college students remains limited. Relevant psychological research further notes that sports achievement emotions and exercise motivation serve as core mediators connecting personal psychological traits to sports consumption decisions (Y. Sun et al., 2025), and students’ mental status and healthy lifestyle habits also shape their valuation of different promotional benefits (Y. Wang et al., 2025), which provides our subsequent interpretation of behavioral and neural findings. But few studies have linked individual cognitive characteristics to consumption preferences, and even fewer have adopted neuroscientific tools to reveal the underlying cognitive neural mechanisms, leaving a research gap.

Decision-making style, as a cognitive trait, directly determines how individuals process information, allocate decision resources, and form preferences (Dewberry et al., 2013). One mainstream strand of existing research classifies decision-making styles into two typical types (Schwartz et al., 2002), maximizing and satisficing, while other scholars adopt alternative classification standards such as the five-category framework proposed by Scott and Bruce (1995). Maximizers tend to invest considerable time and effort in comparing options and conducting in-depth calculations to pursue the optimal outcome (Dar-Nimrod et al., 2009). In contrast, satisficers rely more on intuitive experience and subjective judgment, and they stop searching once they find an option that meets their basic expectations (Hu et al., 2026). Existing literature has confirmed that decision-making styles significantly affect consumer behavior (Bubić & Erceg, 2018). Although Healthy China-related policy and sports media publicity continuously stimulate undergraduates’ sports consumption motivation and participation intention (F. Li, 2025), few existing studies have examined how maximizing versus satisficing traits shape college students’ heterogeneous preferences across no promotion, discount promotion and public welfare promotion. Accordingly, we propose the first hypothesis:

**H1.**  *Maximizers prefer discount promotion, while satisficers favor public welfare promotion*.

A growing number of neuroimaging studies have combined fNIRS technology to uncover the prefrontal neural basis of varied decision-making styles, providing solid methodological support for the present multimodal research design. Balada et al. (2025) adopted fNIRS to monitor prefrontal oxygenation during situational decision tasks and verified that decision-making traits directly alter prefrontal activation amplitude, Y. Li et al. (2019) further confirmed medial prefrontal cortex (mPFC) asymmetric activation differentiates risky versus ambiguous decision preferences via fNIRS recording, and K. Zhou et al. (2026) applied fNIRS in simulated decision experiments and found indecisive cognitive traits correspond to delayed and weakened prefrontal hemodynamic signals. Consistent with Balconi et al. (2025), these fNIRS-based studies uniformly prove that maximizers engaging in thorough option comparison produce prolonged and stronger prefrontal activation, whereas intuition-driven satisficers show limited transient prefrontal activation. Many findings consistently pinpoint the left dorsolateral prefrontal cortex (left DLPFC) as a core brain region subsuming subjective benefit valuation and consumption-related decision evaluation (Keil et al., 2002). Distinct hemodynamic responses within this region can objectively distinguish rational cost calculation from intuitive value judgment, which is why the present study selects the left DLPFC as the primary neural region of interest (ROI) to unpack style-based preference divergence. Drawing on these findings, we put forward the second hypothesis:

**H2.**  *Maximizers’ left DLPFC activates more strongly for discount promotion, and satisficers’ activates more for public welfare promotion*.

Based on existing behavioral and fNIRS evidence reviewed above, this study has three contributions. First, it takes decision-making style as the core explanatory variable to explore the micro cognitive mechanism underlying consumption preferences, supplementing the macro-oriented literature. Second, it combines behavioral experiments with functional near-infrared spectroscopy (fNIRS) to capture both external preference performance and internal brain activation patterns, achieving an interdisciplinary integration of behavioral science and cognitive neuroscience in sports consumption research. Third, it systematically compares preferences across three conditions (no promotion, discount promotion, and public welfare promotion) and clarifies how decision-making styles shape preferences through information processing depth and neural activation modes.

This study employs a one-factor within-subjects design to investigate consumption preferences and left dorsolateral prefrontal cortex activation among maximizers and satisficers under the three promotion conditions. By integrating behavioral and neuroscientific evidence, this study aims to uncover how decision-making styles influence college students’ sports and health consumption choices.

## 2. Method

This study adopted a one-factor within-subjects design, combining behavioral experiments and functional near-infrared spectroscopy (fNIRS) to explore the effects of decision-making styles on college students’ sports and health consumption preferences. A total of 45 non-sports-major undergraduates with badminton consumption experience were initially recruited via public invitation. This is because badminton enjoys high participation rates among university students, and focusing on one single sport avoids confounding effects caused by heterogeneous sport attributes and ensures unified experimental stimuli. The required sample size was calculated by G*Power 3.1 software with a significance level of 0.05 and statistical power of 80% (Faul et al., 2007), via a priori power analysis before participant recruitment. Six participants were excluded due to poor fNIRS signal quality and excessive head movement, leaving 39 valid participants (15 males, 24 females) with a mean age of 23.00 ± 1.52 years. Participants were recruited via purposive sampling targeting full-time undergraduates with regular badminton experience from multiple university majors to avoid single-major bias. Preliminary screening excluded students without badminton consumption experience to guarantee effective experimental manipulation. All participants had normal vision and no central nervous system infection history, signed informed consent, and the study was approved by the university ethics committee.

The experimental scenario was set as badminton stadium consumption, and three types of promotion materials were designed, no promotion, discount promotion, and public welfare promotion, all consisting of unified-style pictures and standardized texts to control irrelevant variables, such as picture layout and font size across all materials. The simulated exercise time was fixed at 6:00 p.m. on Mondays and Wednesdays, and 3:00 p.m. on Sundays, with clear fee rules for each promotion mode (details in Table 1).

A revised 9-item decision-making style scale (7-point Likert) was used to classify participants, which was developed by Yang & Chiou (2010) and Rogge (2017). A small-scale pilot study with 5 undergraduates was completed before formal data collection. Based on participants’ feedback, we slightly modified item wording to adapt the scale for Chinese undergraduates and all nine items are summed into a single total score. The 7-point Likert is anchored as 1 = completely disagree and 7 = completely agree (details in Table 2). The scale showed good reliability (Cronbach’s α = 0.868) and validity (χ^2^/df = 1.505, SRMR = 0.084, RMSEA = 0.115, CFI = 0.943, IFI = 0.947, TLI = 0.902). The correlation coefficients between each item and the total scale score ranged from 0.313 to 0.886, and the 95% confidence intervals of these coefficients did not include 1, indicating satisfactory discriminant validity.

In the study, maximizing decision-making style emphasizes that individuals focus more on the attribute information of products or services and pursue an optimal choice when evaluating decisions. In contrast, satisficing decision-making style highlights that individuals make evaluations based on intuition and pay greater attention to subjective experience during decision-making. According to previous studies (Schwartz et al., 2002; N. Liu et al., 2021), we adopted the sample mean for group classification. In this study, participants scoring above the sample mean (M = 4.20) were categorized as maximizers (*n* = 22), and those scoring below the mean were assigned to the satisficing group (*n* = 17). In addition, participants rated their subjective preference for the three promotion modes accordingly, with a scoring interface for sports consumption preference that was designed using a 7-point rating scale, where 1 represented “strongly not prefer” and 7 represented “strongly prefer”. No other interfering information was presented in the scoring interface to ensure the objectivity of the rating results.

**Figure 1:**
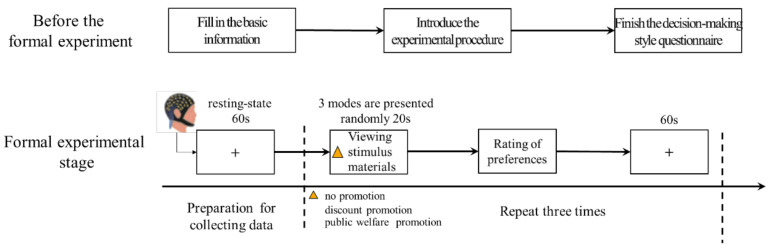
Flowchart of the experiment.

**Figure 2:**
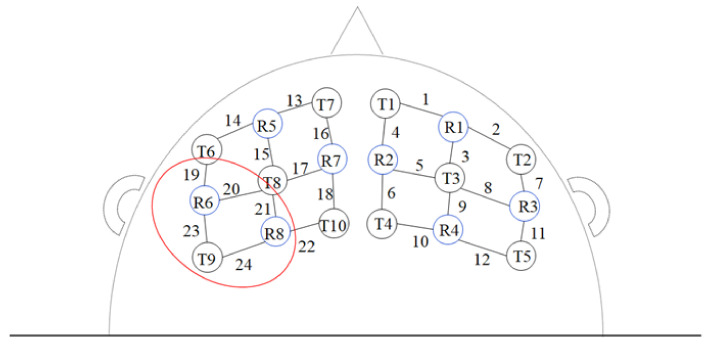
The fNIRS channels.

The experiment was conducted in a quiet laboratory. Participants first provided demographic information, completed the decision-making scale, and wore the fNIRS device with signal calibration. A 60 s resting-state scan was recorded as the baseline. The experiment was programmed with E-Prime 3.0, presenting three promotion stimuli in random order. Each trial included a 5 s fixation, 20 s stimulus viewing, preference rating, and 60 s rest to avoid neural interference. The overall experimental procedure is illustrated in Figure 1. The fNIRS system (Artinis Brite 24) used dual-wavelength detection (762 nm, 841 nm) at 10 Hz sampling rate, with 24 channels covering bilateral dorsolateral prefrontal cortex (DLPFC); channels 19, 20, 21, 23, 24 in the left DLPFC were selected as core regions of interest (ROIs) linked to decision evaluation and benefit perception (Keil et al., 2002) (Figure 2).

fNIRS data were preprocessed via Oxysoft v3.2.7 and MATLAB R2021b, including denoising, spline interpolation, 0.01–0.10 Hz band-pass filtering, and baseline correction, to obtain ΔoxyHb values. Behavioral and neural data were statistically analyzed using SPSS 26.0, with descriptive statistics and one-sample *t*-tests. Given the single-sample design, one-sample *t*-tests were adopted for data analysis.

## 3. Results

Preference ratings and brain activation data were collected from 39 college students under three promotion conditions: no promotion, discount promotion, and public welfare promotion. As shown in Table 3, the overall preference order was public welfare promotion (5.03 ± 1.308) > discount promotion (4.82 ± 1.189) > no promotion (2.95 ± 1.213). Maximizers (*n* = 22) gave the highest rating to discount promotion (5.05), since they calculated total weekly cost and found it more economical than public welfare promotion. Satisficers (*n* = 17) preferred public welfare promotion most (5.12), based on intuitive price perception rather than detailed computation. Post-experiment semi-structured interviews with all 39 participants supported these behavioral patterns: maximizers focused on cost–benefit comparison, while satisficers responded to intuitive preferential signals (Balconi et al., 2025). Accordingly, H1 was supported.

**Figure 3:**
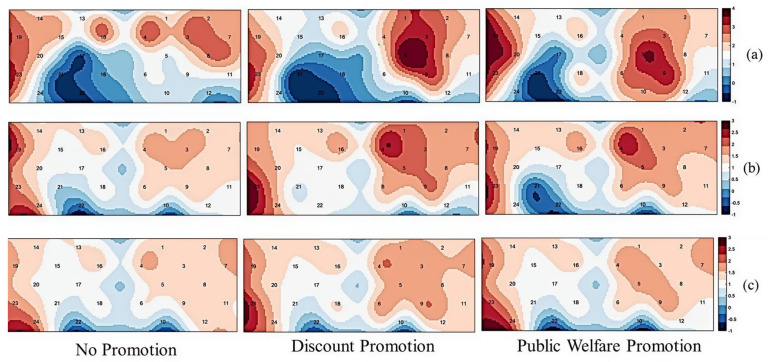
Brain activation images under different modes. (**a**) ΔoxyHb of the whole sample; (**b**) ΔoxyHb of the maximizers; (**c**) ΔoxyHb of the satisficers.

Brain activation was measured by ΔoxyHb in the left dorsolateral prefrontal cortex (DLPFC). As shown in Figure 3a, public welfare promotion induced the strongest and most widespread activation across the whole sample, followed by discount promotion and no promotion. No promotion only weakly activated channel 23. Discount promotion significantly activated channel 23 and mildly activated channel 21. Public welfare promotion strongly activated channels 19, 20, and 23, indicating simultaneous processing of economic benefit and social value. This interpretation is consistent with prior fNIRS evidence that the DLPFC subserves the integration of economic and social values during decision-making (Y. Li et al., 2019; Steinbeis et al., 2012).

Group activation further differed by decision-making style. As shown in Figure 3b, maximizers exhibited the strongest activation in channel 23 under discount promotion (t = 3.87, *p* < 0.001, Cohen’s d = 0.83), with significantly higher ΔoxyHb than under public welfare promotion; activation under public welfare promotion was weaker and limited to fewer channels. As presented in Figure 3c, satisficers showed prominent activation in channels 19 and 20 under public welfare promotion (t = 3.52, *p* < 0.01, Cohen’s d =0.85; t = 3.17, *p* < 0.01, Cohen’s d = 0.77), with stronger overall activation than under discount promotion, which only moderately activated channel 23. These results supported H2.

In summary, behavioral preferences were fully consistent with neural activation patterns: maximizers were more responsive to discount promotion in both rating and brain activity, while satisficers were more strongly driven by public welfare promotion.

## 4. Discussion

This study integrated behavioral experiments and fNIRS neuroimaging to examine how maximizing and satisficing decision-making styles influence college students’ sports and health consumption preferences under three promotional modes. The findings uncovered consistent behavioral and neural differences, clarifying the cognitive and neural mechanisms underlying preference formation.

Behaviorally, the overall preference order was public welfare promotion > discount promotion > no promotion. This pattern reflects college students’ sensitivity to both economic incentives and social value embedded in national fitness policies, which is consistent with previous study highlighting young consumers’ inclination toward prosocial consumption under public health policies (Borusiak & Kucharska, 2022). Influenced by the Healthy China Initiative and national fitness campaigns, college students value both economic gains and prosocial fulfillment, incorporating public welfare and sports values into their daily cognition (Y. Sun et al., 2025). Public welfare promotions let students enjoy low-cost sports access while supporting public sports development, satisfying their altruistic needs (Borusiak & Kucharska, 2022). The preferential pricing of welfare schemes fits students’ constrained budgets and brings greater cost advantages than uniform discounts or original pricing (C. Zhou et al., 2025). And post-study interviews revealed that the positive emotional experience brought by welfare settings also drives their overall preference. Decision-making styles led to clear heterogeneity: maximizers preferred discount promotion because they conducted in-depth cost–benefit calculation to pursue maximum economic returns, while satisficers prioritized public welfare promotion based on intuitive value perception without extensive computation. This distinct preference divergence between two groups is consistent with classic decision-making research (Dar-Nimrod et al., 2009; Hu et al., 2026).

Neurally, the left dorsolateral prefrontal cortex (DLPFC), a region associated with reward evaluation and social cognition (Steinbeis et al., 2012), showed activation patterns consistent with behavioral preferences. Public welfare promotion induced widespread and strong activation, reflecting dual processing of economic benefits and social identity. Discount promotion only triggered localized activation related to quantitative benefit perception. No promotion elicited minimal activation. Moreover, maximizers exhibited the strongest left dorsolateral prefrontal cortex (DLPFC) activation under discount promotion, whereas satisficers showed significantly greater activation under public welfare promotion, revealing the neural basis of divergent decision-making patterns. Such divergent activation patterns align with prior neuroimaging findings on dorsolateral prefrontal cortex (DLPFC)’s core function in reward and social value processing (Balconi et al., 2025).

These results contribute to the literature by highlighting individual cognitive characteristics as key drivers of sports consumption, complementing previous macro-level research. The integration of behavioral and neuroscientific evidence bridges consumer behavior and cognitive neuroscience in the sports domain. Different from existing fNIRS and neuroeconomic studies on general consumer decisions, this research explores decision patterns in sports consumption scenarios under diverse promotional conditions. Practically, the findings support precision marketing for sports venues and evidence-based policy design for national fitness promotion.

## 5. Conclusions, Implications and Future Research Directions

### 5.1. Conclusions

This study explores how maximizing and satisficing decision-making styles affect college students’ sports and health consumption preferences using behavioral experiments and fNIRS, with 39 participants under three promotion conditions. Behaviorally, overall preference ranks public welfare promotion, discount promotion, and no promotion. Maximizers prefer discount promotion via rational cost–benefit calculation for maximum benefits, while satisficers favor public welfare promotion based on intuitive value perception. Neurally, public welfare promotion widely activates the left dorsolateral prefrontal cortex, while discount promotion only triggers local activation; maximizers show strongest activation under discount promotion, and satisficers under public welfare promotion. Decision-making styles shape preferences through information processing depth and brain activation patterns.

### 5.2. Implications

This research supplements micro cognitive evidence for sports consumption research by linking maximizing–satisficing traits with consumption preference, enriching relevant consumer decision literature. Combining behavioral test and fNIRS expands empirical methods in sports consumption and provides new neural evidence for decision-making style research.

Sports venues can carry out precise differentiated marketing: launching targeted discount activities, such as weekly membership discounts and package deals, to attract maximizer groups who focus on cost–benefit. For crowds dominated by satisficers, venues may prioritize public welfare preferential programs, including public welfare open days and low-cost experience activities that emphasize social value and public benefits. Meanwhile, relevant government departments can optimize public sports welfare policies to stimulate youth sports consumption under the Healthy China strategy.

### 5.3. Limitations and Future Research Directions

This study only adopted a single badminton consumption scenario and a relatively small sample of college students, restricting the generalization of research findings. In addition, demographic variables such as gender and monthly disposable income are not included for cross-group interaction analysis.

Follow-up research can expand sport types and enlarge sample size across multi-type universities. Subsequent work can further explore how demographic factors interact with decision-making styles to affect consumption preference; more neuroimaging indicators can also be introduced to reveal underlying brain mechanisms.

## Figures and Tables

**Table 1:** Experimental materials under different promotion modes.

Mode	Image Materials	Text Materials
No Promotion	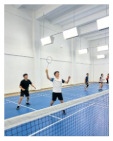	Fee: 30 yuan per 60 min per court area. Personal exercise time: 6:00 p.m. every Monday and Wednesday, and 3:00 p.m. every Sunday.
Discount Promotion	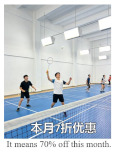	In support of National Fitness Program, a 30% discount is offered this month. Fee: 21 yuan per 60 min per court area (original price is 30 yuan). Personal exercise time: 6:00 p.m. every Monday and Wednesday, and 3:00 p.m. every Sunday.
Public Welfare Promotion	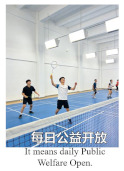	Public welfare opening: Badminton court area fee is 5 yuan per 60 min per court daily from 8:00 to 10:00 a.m. and 2:00 to 4:00 p.m.; other times are 30 yuan per 60 min per court area. Personal exercise time: 6:00 p.m. every Monday and Wednesday, and 3:00 p.m. every Sunday.

**Table 2:** Measurement scale for consumer decision-making styles.

Variable	Item	Source
Decision-Making Style	1. No matter how much effort it takes, I always try to choose the best option.	(Yang & Chiou, 2010; Rogge, 2017)
	2. I do not like settling for “good enough”.	
	3. I am a person who strives for maximization.	
	4. For everything I do, I hold myself to the highest standards.	
	5. I will wait as long as it takes for the best option to appear.	
	6. I am never satisfied with the second-best choice.	
	7. I find it hard to make a decision until I have considered all available options.	
	8. When facing a choice, I imagine all other possibilities, including those that do not yet exist.	
	9. I am never satisfied.	

**Table 3:** Preference ratings for different promotion modes (mean ± standard deviation).

**Participant Preference Ratings**	**N**	**No Promotion**	**Discount Promotion**	**Public Welfare Promotion**
Overall	39	2.95 ± 1.213	4.82 ± 1.189	5.03 ± 1.308
Maximizing group	22	3.19 ± 1.402	5.05 ± 1.253	4.95 ± 1.463
Satisficing group	17	2.65 ± 0.862	4.53 ± 1.068	5.12 ± 1.235

## Data Availability

The raw data supporting the conclusions of this article will be made available by the authors on request.

## Abbreviations

The following abbreviations are used in this manuscript:

fNIRS	functional near-infrared spectroscopy
DLPFC	dorsolateral prefrontal cortex
ROI	region of interest
